# Cardio-metabolic and immunological impacts of extra virgin olive oil consumption in overweight and obese older adults: a randomized controlled trial

**DOI:** 10.1186/s12986-015-0022-5

**Published:** 2015-08-07

**Authors:** Mitra Rozati, Junaidah Barnett, Dayong Wu, Garry Handelman, Edward Saltzman, Thomas Wilson, Lijun Li, Junpeng Wang, Ascensión Marcos, José M. Ordovás, Yu-Chi Lee, Mohsen Meydani, Simin Nikbin Meydani

**Affiliations:** Jean Mayer USDA Human Nutrition Research Center on Aging at Tufts University, 711 Washington Street, Boston, MA 02111 USA; Department of Health and Clinical Sciences, University of Massachusetts, Lowell 3 Solomont Way, Suite 4, Lowell, MA 01854 USA; Institute of Food Science and Technology and Nutrition (ICTAN), Scientific National Research Council (CSIC), Madrid, Spain

**Keywords:** Olive oil, Immune response, Cardio-metabolic, Aging, Obesity

## Abstract

**Background:**

Both aging and obesity are related to dysregulated immune function, which may be responsible for increased risk of infection and also chronic non-infectious diseases. Dietary lipids have been shown to impact immune and inflammatory responses and cardio-metabolic risk factors. No information on the impact of olive oil on immune responses of overweight and obese older adults is available.

**Objective:**

We aimed to determine the effect of replacing oils used in a typical American diet with extra virgin olive oil for 3 months on immune responses and cardio-metabolic risk factors in overweight and obese older adults.

**Methods:**

This was a randomized, single-blinded and placebo-controlled trial in 41 overweight or obese participants (aged ≥ 65) who consumed a typical American diet. Participants in the control (CON, *n* = 21) group were provided with a mixture of corn, soybean oil and butter, and those in the olive oil (OO, *n* = 20) group, with extra virgin olive oil, to replace substitutable oils in their diet. At baseline and 3 months, we measured blood pressure, biochemical and immunological parameters using fasting blood, and delayed-type hypersensitivity (DTH) skin response.

**Results:**

Compared to the CON group, the OO group showed decreased systolic blood pressure (*P* < 0.05), a strong trend toward increased plasma HDL-C concentrations (*P* = 0.06), and increased anti-CD3/anti-CD28 -stimulated T cell proliferation (*P* < 0.05). No differences were found in T cell phenotype, cytokine production, and DTH response between the two groups.

**Conclusions:**

Our results indicate that substitution of oils used in a typical American diet with extra virgin olive oil in overweight and obese older adults may have cardio-metabolic and immunological health benefits. This trial was registered at clinicaltrials.gov as NCT01903304.

## Introduction

Olive oil, which is part of the Mediterranean diet (Med-diet), has been shown to have several health benefits including a capacity to lower the risk of cardiovascular disease (CVD), stroke, and certain forms of cancer. The health benefits of olive oil have been linked mostly to its high content of oleic acid and phenolic compounds [1–4].

It is well known that aging is associated with dysregulated immune function and increased susceptibility to infectious diseases as well as higher incidence of non-infectious diseases such as CVD, dementia, arthritis, diabetes, cancer, and autoimmune disorders [5, 6]. Changes in both cell-mediated immunity and inflammatory responses have been reported with aging. While age-associated changes are observed in almost all aspects of the immune system, a decline in T cell function is believed to be the central defect in immunosenescence [7–10]. Age-related decline in *ex vivo* antigen and mitogen-stimulated T cell proliferation [11, 12] as well as *in vivo* T cell responses to immunization [13], and delayed-type hypersensitivity (DTH) skin response [14] have been reported across all species. The decline in T cell-mediated function is attributed somewhat to phenotypic changes in lymphocytes [15–17], decline in IL-2 production [18] and increase in production of T cell suppressive eicosanoids [19, 20].

On the other hand, aging is associated with chronic inflammation as indicated by higher levels of inflammatory markers such as IL-6 and tumor necrosis factor-alpha (TNF-α), both of which may cause substantial tissue damage and dysfunction [5, 21, 22] and are believed to be key players in the pathogenesis of several diseases including CVD, rheumatoid arthritis (RA), neurodegenerative diseases, and cancer [23, 24].

Similar to aging, obesity is associated with low-grade, chronic inflammation, which is thought to contribute to the development of several inflammatory diseases [25, 26]. Obesity is also shown to impair T cell function and resistance to infection [26, 27]. Evidence in both humans and mice has shown that metabolic tissues in the obese (including adipose, liver, muscle, pancreas and brain) compared to those in lean controls secrete more inflammatory mediators/markers such as TNF-α, IL-6 and C reactive protein [28–32]. Studies have shown that increased adherence to the Med-diet enriched by extra virgin olive oil (EVOO) is associated with lower incidence of obesity [33, 34], hypertension [35] and hyperlipidemia [36].

The limited studies conducted on the immuno-modulatory effect of olive oil have shown inconsistent results [37, 38]. Additionally, the majority of these studies have reported the anti-inflammatory and antioxidant effects of olive oil [39–42] with little information available on the impact of olive oil on T cell-mediated immune response in humans. Moreover, few studies have evaluated olive oil’s effect on both T cell-mediated function and inflammatory responses, and none of them have been conducted in overweight or obese older adults. Given that both obesity and aging are associated with increased inflammation and impaired T cell function, it is critical to determine the health benefits of olive oil in this particular population. Further, all previous studies focusing on immune function have used refined olive oil rather than EVOO, which contains phenolic compounds that have antioxidant properties not found in refined olive oil. These components have been suggested to be key factors contributing to EVOO’s beneficial effects [1–3].

There is growing evidence that olive oil has some beneficial effects on CVD through different mechanisms including effects on lipid profile, blood pressure, inflammation, and arterial wall function [35, 36, 43–45]; but to our knowledge, none of those studies have been conducted in overweight or obese older adults. Thus, the current study was conducted to evaluate whether substituting oils in a typical American diet with EVOO would improve T cell-mediated immune function and inflammatory responses as well as factors related to cardio-metabolic status in overweight or obese older adults such as lipid profile and blood pressure.

### Subjects and methods

#### Participants

Participants for this study were recruited by the Recruitment and Volunteer Services Department at the Jean Mayer USDA Human Nutrition Research Center on Aging (HNRCA) at Tufts University by inviting individuals within the specified age and body mass index (BMI) ranges in the HNRCA recruitment database, advertising in various local newspapers, in media sources, at the Tufts University Boston campus, Tufts Medical Center clinics, and on public bulletin boards in the downtown Boston area and neighboring towns. A total of 960 responses were received. After telephone pre-screening, 799 individuals were considered ineligible because they either were no longer interested or did not meet study criteria. Following laboratory screenings, an assessment of medical history and a physical examination performed by a study nurse practitioner in the Metabolic Research Unit (MRU) at the HNRCA, an additional 117 of the remaining 161 individuals were found ineligible to participate.

Subjects were excluded if they reported that they did not speak English, were blind or deaf, were on a vegetarian diet, ate more than 3 meals per week at a restaurant, had chronic eating disorders, were HIV+, or had autoimmune diseases or cancers (except for non-melanoma skin cancer), used chemotherapy or immunosuppressive drugs, or had any major illnesses including uncontrolled CVD, liver disease, renal disease, diabetes, hypertension, asthma, a history of splenectomy, were on dialysis or had any conditions associated with maldigestion or malabsorption including pancreatitis, celiac disease, gastric bypass or surgery for weight loss. Additionally, they were excluded if they were diagnosed with or were in treatment for psychosis and had obtained a BDI-II (Beck Depression Inventory-II) score ≥ 20 or MMSE (Mini Mental State Examination) score ≤ 25. Finally, they were excluded if they consumed more than 2 glasses of alcoholic drinks/day or had a history of smoking; further, they were excluded if any of the following occurred prior to study blood draws and skin tests: they had used nicotine within 6 months, were on antibiotics or had infections within 2 weeks, received flu vaccination within 3 weeks and tetanus immunization within 6 weeks.

Subjects of both genders were included if they were 65 y or older, had a BMI between 25–35 kg/m^2^, were willing to stop using dietary supplements (except vitamin D and calcium) including multivitamin and minerals, fish oil, olive oil, and canola oil, 30 days before and during the study. Additionally, they were consuming and were required to continue consuming a typical American diet. The diet composition of the typical American diet has been determined by National Health and Nutrition Examination Survey (NHANES) as follows: carbohydrates make up 50 % of daily energy requirements and include refined and processed grain products, fruits and vegetables and dairy products. Protein constitutes 15 % of daily energy requirements provided from sources such as meats and dairy products. Fat makes up 35 % of daily energy requirements, which represents 86 g of fat/day in a 2200 calorie diet. The fatty acid composition includes polyunsaturated fatty acids (PUFA), saturated fatty acid (SFA), and monounsaturated fatty acids (MUFA) at the ratio of 1:2:2. Dietary fiber and cholesterol intake are 12–14 g/day and ≥400 mg/day, respectively [46, 47].

**Fig. 1:**
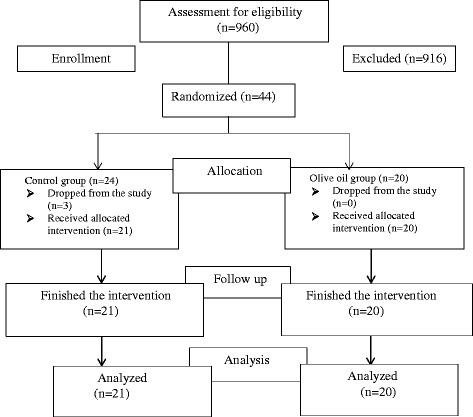
Study profile for recruitment and enrollment

The forty-four subjects who were eligible to participate in the study signed the informed consent form, and 41 participants completed the study. Three (3) participants dropped out of the study: one was prescribed an iron supplement, one refused to have DTH skin test implant, and one had an accident unrelated to the study (Fig. 1).

#### Study design

We conducted a randomized, single-blinded, and placebo-controlled trial in overweight and obese older adults between 2011 and 2013 to determine the impact of replacing substitutable oils in a typical American diet with extra virgin olive oil. The study protocol and consent form were approved by the Tufts University/Tufts Medical Center Institutional Review Board. Eligible participants were randomized into either the CON or OO group in a 1:1 ratio.

The coding for the CON and treatment groups was assigned by the study statistician, who had no contact with subjects and had no role in data collection. The study oils (CON or OO) were labeled with the subjects’ names by the study dietitian, who also held the randomization code; all other investigators and the study nurses were blinded to this information. Participants, however, could not be blinded since they might recognize the smell and taste of EVOO.

The oils were provided in a bottle or as a spread. To minimize bias, both study oils were provided to participants in the same type of bottle (oil) and the spread in the same type of container (spread). The CON oil was a blend of 10 % corn oil and 90 % soybean oil, and the CON spread was butter; these were defined as the oils consumed in a typical American diet [48]. The EVOO used for the study was provided by the Deoleo Company in Cordoba, Spain. Prior to screening, participants were asked to taste the oil/spread and to confirm they were willing to consume these oils while on the study (Table 1 shows the composition of fatty acids in both the CON and OO groups).

**Table 1:** Composition of fatty acids in control and olive oils/spreads

Fatty acids	Olive oil	Control oil	Butter (control spread)
		%	
C14:0	0.4	0.1	11.5
C16:0	13.5	10.9	36.2
C16:1	1.3	0	0
C18:0	2.4	4.4	15.1
C18:1n9	68.3	22.7	30.7
C18:1n7	3.2	1.4	1.9
C18:2n6	10	54.2	4.1
C18:3n6	0	0	0
C18:3n3	0.7	6.3	0.5
C20:1n9	0.3	0	0
C20:4n6	0	0	0
C20:5n3	0	0	0
C22:5n6	0	0	0

Fatty acids (%) in control and olive oil samples analyzed by gas chromatography as described in method section

Participants consumed the assigned oils for 3 months. During the study, both groups continued their typical American diet with only one change: they replaced substitutable oils in their diet (cooking oil, spread, and oils in dressings) with the study oil/spread provided to them. The study oils were distributed to the participants by the dietitians at the MRU of the HNRCA.

#### Baseline and month 3 study visits

Subjects were asked to come to the center for 4 consecutive days at the beginning (baseline) of the study and 3 months after enrollment. On day 1, vital signs (temperature, blood pressure, pulse, and respiratory rate), weight, height, and waist and hip circumferences were measured. Subjects were asked to discontinue anti-inflammatory medicines including aspirin or anti-histamines 72-h before each blood collection until 48-h after DTH implantation. On day one, blood was collected after a 12-h fast for blood chemistry, lipid profile, complete blood count (CBC) differential, fatty acids, and immunological tests described below. On day 2, a second blood sample was drawn for repeated *ex vivo* immune tests. Then, three (3) recall antigens (detailed below) and saline were implanted in the forearm of each subject for the DTH skin test, as an *in vivo* test of cell-mediated immune function [49]. On day 3 and 4, participants visited the MRU for an evaluation of their DTH skin response at 24-h and 48-h post-administration, respectively.

#### Diet intervention visits and assessment of dietary intake

Dietitians at the MRU at HNRCA provided participants with instructions for substituting fats used in their diets with study oils and spreads. Dietary counseling was provided every two weeks or more often as needed to ensure that all subjects were following the instructions, were using the right oil and adhering to the appropriate diet. Compliance with consuming study oils was evaluated by the dietitians using a “checklist of study oils consumed per week” as indicated by participants when they returned used containers for a new supply of study oils and spreads as well as by plasma fatty acid analysis (see below). To assess dietary intake, participants completed a 3-day dietary record (3DDR) at both baseline and month 3, which were reviewed and analyzed by a dietitian using the Minnesota Nutrient Data System (Nutrition Coordinating Center, University of Minnesota, Minneapolis, MN), version 2010.

#### CBC differential, blood chemistry, and blood lipid profile

Fasting blood was used for evaluation of CBC-differential [50], blood glucose [51], and blood lipid profile [triglycerides [52], total cholesterol [53], low density lipoprotein cholesterol (LDL-C), and high density lipoprotein cholesterol (HDL-C) [54] at baseline and month 3 by the Nutritional Evaluation Laboratory (NEL) at the HNRCA.

#### Fatty acid analysis

Compliance of study oil intake was determined by measuring fatty acid composition in the plasma of participants by gas chromatography (GC) method [55].

#### Immunological assessment

##### Lymphocyte phenotype

To determine lymphocyte phenotype, whole blood surface staining of different white blood cell markers was done using fluorescent antibodies (all from eBioscience, San Diego, CA) for total T cells (CD3+), helper T cells (CD4+), cytotoxic T cells (CD8+), naïve and memory subpopulations within CD4 and CD8 T cells (determined by their combined expression of CD45RA, CD45RO, and CD62L), B cells (CD19+), and natural killer T cells (CD3 + NK+). Isotype staining was also done as the negative control. Flow cytometric measurement was conducted using an Accuri C6 flow cytometer (BD Biosciences, San Jose, CA). Data were analyzed using Flowjo 7.6 software (TreeStar Inc., Ashland, OR).

##### Lymphocyte proliferation

T cell proliferation was measured by [^3^H]-thymidine incorporation after stimulation of whole blood culture with different stimuli described below. To account for the day-to-day variability in *ex vivo* immunological measurements, blood was collected two times (day 1 and day 2), and the average of the two measurements was used in statistical analysis. Heparinized blood was diluted 1:10 (final v: v) with RPMI 1640 supplemented with 100 kU/L penicillin, 100 mg/L streptomycin, 2 mmol/L L-glutamine, and 25 mmol/L HEPES (Gibco Laboratories, Grand Island, NY). Diluted blood was stimulated with T cell mitogen concanavalin A (Con A, Sigma, St. Louis, MO) at 5, 25, and 50 mg/L, or immobilized anti-CD3 (eBioscience) at 1, 5, and 10 mg/L and soluble anti-CD28 (1 mg/L), and incubated in 96-well round-bottom cell culture plates for 72-h. Cultures were pulsed with 0.5 μCi [^3^H]-thymidine during the last 4-h of incubation. Cells were harvested onto glass fiber mats using a Perkin Elmer cell harvester (model No. C961961, Waltham, MA). Cell proliferation was quantified as the amount of [^3^H]-thymidine incorporation into DNA, which is determined by liquid scintillation counting in a Perkin Elmer counter (model No. 2450–0060). T cell proliferation is expressed as count per minute (cpm).

##### Cytokine and PGE_2_ production

Heparinized whole blood was diluted 1:4 (final v: v) with RPMI 1640. To determine T cell cytokine IL-2 and IFN-γ production, diluted whole blood was stimulated with Con A (25 mg/L) or immobilized anti-CD3 (5 mg/L) and soluble anti-CD28 (1 mg/L) in 24-well plate for 48-h. In addition, we also assessed the impact of olive oil on inflammatory response. Diluted whole blood was stimulated with lipopolysaccharide (LPS) (1 mg/L) in 24-well plates for 24-h to measure the production of pro-inflammatory cytokines IL-6 and TNF-α as well as lipid inflammatory mediator PGE_2_ using ELISA.

For all cytokines including IL-2, IFN-γ, IL-6, and TNF-α, reagents were from BD Bioscience. For PGE_2_, high sensitivity enzyme immune assay (EIA) kit (ENZO Life Science Inc, PA, USA) was used. Plates were read in a micro-plate reader (BioTek, model No. EL808) and analyzed by Gene5 software.

##### Delayed-type hypersensitivity (DTH) skin test

Three recall skin test antigens, Candida Albicans (Candin, Allermed Laboratories Inc, San Diego, CA), Tetanus Toxoid (Toxoid Adsorbed, Sanofi Pasture, CA), and Trichophyton Mentagrophytes (Trichophyton, Allermed Laboratories Inc, San Diego, CA) were injected intradermally at separate sites on the volar surface of the forearm. Saline (0.9 %) was used as the negative control. Candida, Trichophyton and saline were injected in a standard volume of 0.1 ml, and tetanus toxoid was injected in a volume of 0.025 ml. Skin tests were read 24 and 48-h after antigens were applied; a test was considered positive if the skin induration was ≥ 5 mm.

#### Statistical analysis

The demographic characteristics and other variables of interest of the participants in the CON versus OO group were compared at baseline by using Student’s *t*-test or Chi-square test. Paired *t*-test or nonparametric test was performed to compare the measures at baseline and month 3 within each group. Differences in outcome measures of interest between the groups were determined using Analysis of Covariance (ANCOVA) controlling for their corresponding baseline levels, sex and age or using non-parametric test. We used log transformation for the data that were not normally distributed. Two-sided significance at *P* < 0.05 was considered to be significant. Analyses were performed using IBM SPSS Statistics version 21.

### Results

#### Assessment of dietary and macronutrients intake

The average amount of study oil and spread consumption was similar in the CON and the OO group (Table 2). We assessed compliance by conducting both dietary assessment (Table 3) and plasma fatty acid analysis (Table 4). No difference was found in macronutrient intake at baseline between groups. The OO group showed an increase of total monounsaturated fatty acids (MUFA) (*P* < 0.001) and oleic acid (*P* < 0.001) consumption compared to the CON group, and the CON group had increased polyunsaturated fatty acids (PUFA) intake (*P* < 0.05) compared to the OO group after 3 months. No difference in intake of other macronutrients including total fat, carbohydrates, protein, fiber, and energy was observed between the groups after 3 months.

**Table 2:** Study oil intake of the study subjects^a^

	Control oil group	Olive oil group	
Intake	(*N* = 19)	(*N* = 18)	*P* ^*^
Provided oil/spread (g/day)	41 ± 8	39 ± 7	0.34
Energy (Kcal/day)	338 ± 69	347 ± 61	0.41
Total fat (g/day)	38 ± 8	39 ± 7	0.41
Total SFA (g/day)	11 ± 2	5 ± 1	<0.05
Total MUFA (g/day)	9 ± 2	29 ± 5	<0.05
Total PUFA (g/day)	17 ± 4	4 ± 1	<0.001

Abbreviations: *SFA* saturated fatty acids, *MUFA* Monounsaturated fatty acids, *PUFA* Polyunsaturated fatty acids

^*^ P-value was obtained by unpaired *t*-test

^a^ Values are mean ± SEM

**Table 3:** Dietary intake of overweight and obese older adults in control and olive oil groups at baseline and month 3^a^

	Control (*N* = 19)		Olive Oil (*N* = 18)		
Intake/day	Baseline	Month 3	Baseline	Month 3	*P* ^*^
Energy intake (Kcal)	1921 ± 130	1956 ± 142	1781 ± 128	2032 ± 104	0.31
Carbohydrate (g)	231 ± 17	217 ± 18	221 ± 21	225 ± 19	0.51
Carbohydrate (% Kcal)	47 ± 1	43 ± 2	47 ± 2	43 ± 2	0.75
Protein (g)	78 ± 6	72 ± 6	75 ± 5	82 ± 6	0.16
Protein (% Kcal)	17 ± 1	15 ± 1	17 ± 1	16 ± 1	0.31
Total dietary fiber	23 ± 3	22 ± 3	22 ± 3	22 ± 2	0.72
Fat (g)	77 ± 7	91 ± 9	68 ± 6	90 ± 6	0.66
Fat (% Kcal)	35 ± 1	41 ± 3	34 ± 3	39 ± 2	0.79
Total SFA (g)	23 ± 2	28 ± 3	23 ± 2	25 ± 2	0.33
Total MUFA (g)	28 ± 3	29 ± 3	24 ± 2	44 ± 4	<0.001
C18:1n9 (g)	27 ± 3	27 ± 3	23 ± 2	42 ± 4	<0.001
Total PUFA (g)	20 ± 2	28 ± 4	16 ± 2	15 ± 1	0.02

Abbreviations: *MUFA* monounsaturated fatty acids, *PUFA* polyunsaturated fatty acids, *SFA* saturated fatty acid

^*^ *P*-value for difference between two groups was obtained by ANCOVA adjusting for age, sex and baseline value

^a^Values are mean ± SEM

**Table 4:** Plasma fatty acids of overweight and obese older adults in control and olive oil groups at baseline and month 3^a^

	Control (*N* = 20)			Olive Oil (*N* = 20)		
Fatty Acids	Baseline	Month 3		Baseline	Month 3	*P* ^*^
			%			
C18:1n9 (OA)	19 ± 1	18 ± 1		19 ± 1	20 ± 1	0.04
Total n-3	5 ± 1	5 ± 1		4 ± 0.3	4 ± 0.3	0.38
Total n-6	43 ± 1	42 ± 1		43 ± 1	41 ± 0.3	0.31
Total PUFA	47 ± 1	47 ± 1		47 ± 1	45 ± 1	0.04
Total MUFA	22 ± 1	22 ± 1		22 ± 1	24 ± 1	0.04
Total SFA	31 ± 0.4	31 ± 1		31 ± 1	32 ± 1	0.47
OA/PUFA (ratio)	0.4 ± 0.02	0.4 ± 0.02		0.4 ± 0.02	0.5 ± 0.02	0.03
n-6/n-3 (ratio)	13 ± 1	12 ± 1		12 ± 1	12 ± 1	0.58
ALA	0.6 ± 0.03	0.6 ± 0.04		0.6 ± 0.04	0.6 ± 0.1	0.45

Abbreviations: *ALA* alpha linolenic acid, *MUFA* monounsaturated fatty acids, *OA* oleic acid, *PUFA* polyunsaturated fatty acids, *SFA* saturated fatty acids

^*^ *P*-value for difference between two groups was obtained by ANCOVA adjusting for age, sex and baseline value

^a^Values are mean ± SEM

Plasma fatty acid analysis (Table 4) indicated that participants in the OO group had increased plasma oleic acid (*P* < 0.05), total MUFA (*P* < 0.05), and oleic acid/PUFA ratio (*P* < 0.05) compared to the CON group after 3 months. In addition, there was a reduction in total plasma PUFA in the OO group compared to the CON group after 3 months (*P* < 0.05).

#### Demographic, anthropometrics, blood pressure, blood chemistry, and lipid profile

There was no significant difference in age, gender and race between the two groups at baseline (Table 5). We observed a reduction in systolic blood pressure in the OO group compared to the CON group (*P* < 0.05) after 3 months (Table 5). There was no difference in other anthropometric measures between the two groups at baseline or after 3 months of intervention. In addition, participants in the OO group showed a trend (*P* = 0.06) toward an increase in plasma HDL-C levels compared to the CON group after 3 months (Table 5). No difference was found in other parameters of lipid profiles tested including total cholesterol, LDL-C, and triglycerides as well as in blood glucose concentration between the two groups at month 3.

**Table 5:** Demographic, anthropometric and cardio-metabolic measures of overweight and obese older adults in control and olive oil groups at baseline and month 3^a^

	Control (*N* = 21)		Olive Oil (*N* = 20)		
Measures	Baseline	Month 3	Baseline	Month 3	*P* ^*^
Age (y)	72 ± 1		72 ± 1		0.77
Female, [n (%)]	15 (71)		12 (60)		0.52
Caucasian, [n (%)]	15 (71)		13 (65)		0.57
Weight (Kg)	80 ± 2	80 ± 2	80 ± 3	80 ± 3	0.39
Height (m)	1.7 ± 0.02	1.7 ± 0.02	1.7 ± 0.02	1.7 ± 0.02	N/A
BMI (Kg/m2)	29 ± 1	29 ± 1	29 ± 1	29 ± 1	0.31
Waist (cm)	97 ± 2	97 ± 2	97 ± 3	97 ± 3	0.96
Hip (cm)	106 ± 2	107 ± 2	106 ± 2	106 ± 2	0.10
DBP (mmHg)	76 ± 2	73 ± 2	76 ± 2	73 ± 1	0.99
SBP (mmHg)	126 ± 2	126 ± 3	128 ± 3	122 ± 2	0.04
Glucose (mg/dl)	102 ± 2	105 ± 2	101 ± 2	104 ± 2	0.86
HDL-C (mg/dl)	53 ± 2	52 ± 2	52 ± 3	54 ± 3	0.06
LDL-C (mg/dl)	144 ± 7	145 ± 7	130 ± 7	132 ± 6	0.39
TG (mg/dl)	104 ± 10	110 ± 11	103 ± 9	113 ± 13	0.69

Abbreviations: *BMI* body mass index, *DBP* diastolic blood pressure, *HDL-C* high density lipoprotein-cholesterol, *LDL-C* low density lipoprotein cholesterol, *SBP* systolic blood pressure, *TG* triglycerides, *N/A* not applicable

^*^ P-value between groups was obtained by student (un-paired) *t*-test or chi-square (demographic measures) or ANCOVA adjusting for age, sex and baseline value (anthropometric and cardio-metabolic measures)

^a^ Values are mean ± SEM or n (%)

#### Immunological assessments

##### CBC-differential and lymphocyte phenotypes

No difference was found in total number of white blood cells, percent or total number of lymphocytes, monocytes, neutrophils, eosinophils, and basophils at baseline or after 3 months of intervention between the two groups (data are not shown). There was no difference in the changes between the two groups in any of the blood lymphocyte phenotypes measured over 3 months (Table 6).

**Table 6:** Lymphocyte phenotypes of overweight and obese older adults in control and olive oil groups at baseline and month 3^a^

	Control (*N* = 15)			Olive Oil (*N* = 16)		
Cell type	Baseline	Month 3		Baseline	Month 3	P*
			%			
Total CD4+	53 ± 3	56 ± 2		53 ± 3	56 ± 3	0.92
Total CD8+	16 ± 2	15 ± 2		16 ± 2	15 ± 2	0.45
CD4 naïve	34 ± 3	32 ± 4		31 ± 5	30 ± 5	0.82
CD4-CM	42 ± 2	44 ± 2		35 ± 5	38 ± 5	0.99
CD4-EM	19 ± 1	19 ± 1		16 ± 2	16 ± 2	0.77
CD4-TEM	6 ± 1	5 ± 1		5 ± 1	4 ± 1	0.31
CD8 naïve	25 ± 3	27 ± 3		30 ± 4	30 ± 4	0.45
CD8-CM	8 ± 1	8 ± 1		7 ± 1	9 ± 1	0.31
CD8-EM	14 ± 1	15 ± 1		12 ± 2	14 ± 2	0.39
CD8-TEM	50 ± 3	51 ± 3		49 ± 4	47 ± 5	0.42
CD3+	63 ± 3	62 ± 3		60 ± 3	59 ± 4	0.77
CD19+ (B cell)	15 ± 2	15 ± 1		14 ± 2	14 ± 1	0.67
NKT cell	10 ± 1	10 ± 1		10 ± 1	13 ± 2	0.20

Abbreviations: *CM* central memory, *EM* effector memory, *TEM* terminal effector memory, *NKT* natural killer T cell

*P-value for difference between groups was obtained by ANCOVA adjusting for age, sex and baseline value

^a^Values are mean ± SEM

##### Lymphocyte proliferation

We found significant increase in T cell proliferation in response to anti-CD3/anti-CD28 (1 mg/L) after 3 months in the OO group compared to the CON group (*P* < 0.05). There was no difference in T cell proliferation in response to Con A between the two groups (Table 7).

**Table 7:** Proliferative response of T cells to Con A and anti-CD3/anti-CD28 of overweight and obese older adults in control and olive oil groups at baseline and month 3

	Control (*N* = 21)			Olive Oil (*N* = 20)		
Stimulation condition	Baseline	Month 3		Baseline	Month 3	*P* ^*^
			cpm x 10^3^			
Con A-5 mg/L	21 ± 5	24 ± 3		18 ± 3	21 ± 4	0.93
Con A-25 mg/L	31 ± 7	41 ± 5		31 ± 4	39 ± 5	0.61
Con A-50 mg/L	23 ± 5	28 ± 3		21 ± 3	31 ± 4	0.48
Anti-CD3/anti-CD28-1 mg/L	23 ± 5	22 ± 5		19 ± 4	29 ± 4	0.02
Anti-CD3/anti-CD28-5 mg/L	33 ± 7	35 ± 5		32 ± 5	40 ± 5	0.17
Anti-CD3/anti-CD28-10 mg/L	33 ± 7	36 ± 5		31 ± 5	41 ± 5	0.19

Abbreviations: *Con A* concanavalin A, *cpm* counts per minute

^*^ *P*-value for difference between groups was obtained by ANCOVA adjusting for age, sex, and baseline value

##### Cytokine production

**Fig. 2:**
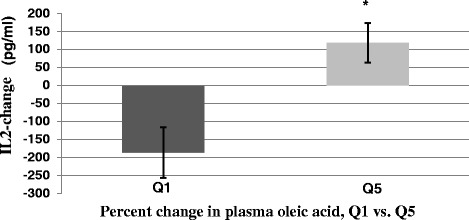
Association between changes in plasma oleic acid content (%) and IL-2 production (all participants). The percent changes in plasma oleic acid were ranked in 5 quintiles, Q1 (−5.14 to-2.68); Q2, (−2.69 to −0.35); Q3, (−0.18 to 0.5); Q4, (0.89 to 2.66), and Q5, (2.78 to 7.32); *N* = 8 participants in each quintile. The change in IL-2 production in response to anti-CD3/anti-CD28 after 3 months is shown in Q1 and Q5. * Significantly different from Q1 by ANCOVA adjusting for age, sex and the baseline values of oleic acid and IL-2 at *P* < 0.01

No difference was found in T cell cytokine and pro-inflammatory cytokine production between the two groups after 3 months of intervention (Table 8). There was heterogeneity in the change of plasma oleic acid concentrations following intervention in both groups. For the CON group, the range of change in plasma oleic acid was between −5.14 and 3.15 %; in the OO group, the range of change was between −3.84 and 7.32 %. This may be partly due to inter-individual variability in endogenous oleic acid production and difference in compliance. As such, we ranked the change in blood oleic acid levels between baseline and month 3 in all participants in quintiles and evaluated the change in cytokine levels between baseline and month 3 in relation to the quintiles 1 and 5 of plasma oleic acid changes. We found that participants who had the highest quintile of oleic acid changes had a higher increase in IL-2 production compared to the participants in the lowest quintile (*P* < 0.01) (Fig. 2). No such association with other cytokines was noted.

**Table 8:** Cytokine concentrations of overweight and obese older adults in control and olive oil groups at baseline and month 3^a^

	Control (*N* = 21)			Olive Oil (*N* = 20)		
Variables	Baseline	Month 3		Baseline	Month 3	*P* ^*^
			pg/ml			
IL-6	28159 ± 2555	26695 ± 3141		28798 ± 2217	30394 ± 3285	0.25
PGE_2_	5264 ± 802	4218 ± 562		3768 ± 605	3593 ± 3593	1.00
TNF-α	1878 ± 269	1806 ± 242		1720 ± 295	1785 ± 200	0.31
IL-2-CD3	218 ± 40	195 ± 41		209 ± 33	236 ± 46	0.73
IL-2-Con A	5709 ± 903	5553 ± 836		6073 ± 690	5500 ± 570	0.78
IFN-γ-CD3	7913 ± 1483	7718 ± 1474		5298 ± 1501	7146 ± 1922	0.11
IFN-γ-Con A	16648 ± 2857	13910 ± 3232		15530 ± 3090	14551 ± 2847	0.20

Abbreviations: *Con A* cancanavalin A, *IFN-γ* interferon gamma, *IL-6* interleukin-6, *IL-2* interleukin-2, *PGE*_*2*_ prostaglandin E_2_, *TNF-α* tumor necrosis factor-alpha

^*^ *P*-value for difference between groups was obtained by ANCOVA adjusting for age, sex, and baseline value

^a^Values are mean ± SEM

##### Delayed-type hypersensitivity skin test (DTH)

Both the CON and OO groups showed an increase in the diameter of skin induration after 24 and 48-h of DTH implant at month 3 compared to baseline, but the increase was similar between the two groups (Table 9).

**Table 9:** Delayed-type hypersensitivity skin test of overweight and obese older adults in control and olive oil groups at baseline and month 3^a^

	Control (*n* = 21)				Olive Oil (*N* = 20)			
Measurement	Baseline	Month 3	*P* ^*^		Baseline	Month 3	*P* ^*^	*P* ^**^
				mm				
Induration to candida	9.4 ± 2.1	13.5 ± 2.4	0.04		9.9 ± 1.9	11.8 ± 2.7	0.13	0.48
Induration to TT	14.6 ± 1.7	16.0 ± 2.5	0.57		10.2 ± 1.9	11.5 ± 1.9	0.73	0.32
Induration to TM	2.6 ± 1.2	4.0 ± 2.5	0.15		4.3 ± 1.6	6.7 ± 2.3	0.07	0.81
Total induration	26.6 ± 2.7	33.5 ± 3.9	0.03		24.4 ± 3.3	30.0 ± 4.4	0.04	0.37
No. of positive responses	1.66	1.86	0.16 ^a^		1.79	1.74	0.74 ^b^	0.08 ^b^

Abbreviations: *TT* tetanus toxoid, *TM* trichophyton mentagrophytes

^*^ *P*-value within group was obtained by paired *t*-test or nonparametric test as indicated by ^b^ under *P* ^*^ column

^**^ *P*-value for difference between groups was obtained by ANCOVA or nonparametric test as indicated by ^b^ under *P* ^**^ column

^a^Values are mean ± SEM or (n) of measurements 48 h after administration of DTH

## Discussion

To our knowledge, our study is the first comprehensive one to determine the impact of substituting extra virgin olive oil for the cooking oils used in a typical American diet on cardio-metabolic and immunological outcomes in overweight and obese older adults, a population at higher risk for infectious and non-infectious chronic diseases. We showed that consumption of extra virgin olive oil for 3 months reduced blood pressure, tended to increase plasma HDL-C levels, and improved T cell proliferation. Further, participants in both groups with the highest change in plasma oleic acid showed higher increase in IL-2 production. There was no effect on other blood lipids, glucose, and inflammatory mediators.

In agreement with the previous studies [35, 56–59], we observed a blood pressure-lowering effect of EVOO consumption. However, our observation is particularly important because the participants, obese and older adults, are at higher risk compared to lean younger adults for developing cardiovascular disease. Furthermore, since lower blood pressure is a predictor of survival and freedom from physical and cognitive impairment [60], both of which increase with age, our results support the notion that consumption of EVOO may have the potential to extend health span and curb development of age-related diseases.

We observed a trend toward increased blood concentrations of HDL-C after olive oil consumption, which supports the earlier reports of a beneficial effect of olive oil on blood lipid profiles by Marrugat et al. [61]. The less pronounced effect observed in our study compared to theirs might be related to the difference in administering olive oil between the two studies. Throughout their study, subjects were provided with a daily dose of 25 mL of raw virgin olive oil and were directed to replace all other cooking fats with refined olive oil. In our study, EVOO was provided to the subjects to use as they wished (reflecting their actual lifestyle); therefore, they might have consumed less uncooked olive oil. Given that heating EVOO might destroy some of its active phenolic compounds [62, 63], it is possible that subjects in Marrugat’s study consumed more uncooked virgin olive oil, and thus, higher levels of phenolic compounds, which might have resulted in a more profound effect on their plasma HDL-C levels.

Limited information is available about the impact of olive oil on immune and inflammatory responses, and no studies have used outcome variables as specific and comprehensive as those in our current study or in any study of overweight and obese older adults. For example, a study by Yaqoob et al. showed no effect on T cell proliferation in healthy middle-aged men (45–64 y, BMI 21.9–30.7) after they consumed only refined olive oil for 8 weeks [37]. This is in contrast to our observation that extra virgin olive oil consumption resulted in a modest improvement in T cell proliferation as well as an association between an increased production of T cell cytokine IL-2 and the magnitude of increase in plasma oleic acid. The difference between our findings and those of Yaqoob et al. [37] might be related to the fact that the participants in our study were 65 y or older, while the age range in their study was 45–64 y. It is reasonable to assume that T cell function in the participants of our study might have been less vigorous due to our subjects’ more advanced ages compared to those in the study by Yaqoob et al. [37], possibly explaining the discrepancy between the results of the two studies. This speculation is also supported by the observation that a study conducted in patients with rheumatoid arthritis, who are known to have reduced T cell-mediated function [64, 65], showed that olive oil increased T- lymphocyte proliferation in peripheral blood mononuclear cells (PBMC) [38]. Additionally, in our study, we used EVOO, which has a higher content of phenolic compounds and might have caused a different effect compared to the refined olive oil used in the study by Yaqoob et al. [37]. Furthermore, the duration of the two studies differed--our study was 12-weeks, their study was 8-weeks. This variation may have also contributed to the difference in findings between the two studies.

To determine the mechanism of olive oil-induced enhancement in anti-CD3/anti-CD28-induced T cell proliferation, we evaluated the production of IL-2, the key cytokine involved in the activation of T cells. When assessing the entire group, we found no difference in IL-2 production between the two study groups; however, since the range of change in plasma oleic acid in the subjects who consumed olive oil (−3.84 % to 7.32 %) and control oil (−5.14 % to 3.15 %) was large, we explored the relationship between quintiles of change in IL-2 production and those in plasma oleic acid in all subjects. Participants with the largest change in plasma oleic acid levels, i.e., those in quintile 5, showed a highly significant increase in IL-2 production compared to those with the lowest change in plasma oleic acid levels, i.e., those in quintile 1. These data suggest that the increase in T cell proliferation might be partly due to an oleic acid-induced increase in IL-2 production. However, further investigation is needed to confirm this finding. Furthermore, our data indicate that the olive oil-induced enhancement in T cell proliferation is not due to changes in blood composition of lymphocytes or T cell profiles as we did not find any difference in the percent of lymphocytes or T cell phenotype profiles between the two groups after 3 months.

It is noteworthy that the effect of olive oil on T cell proliferation in our study may be stimulation-specific because enhanced T cell proliferation was found when anti-CD3/anti-CD28 Abs (TCR activation together with co-stimulation), but not when T cell mitogen Con A, was used to activate T cells. Although mechanisms of signaling pathways were not the subject of our current study, this finding implies that specific signaling pathways or lipid/protein microenvironment involved in T cell receptor activation and co-stimulation-induced T cell activation might be influenced by olive oil.

In contrast to a moderate effect on T cell proliferation, olive oil consumption showed no effect on DTH skin test, an *in vivo* marker for recall antigen-specific cell-mediated immunity. Given that both groups showed an increase in total diameter of induration, it is possible that the boosting effect of repeated administration of the skin test might have masked any additional effect of olive oil.

There are some limitations to this study. First, since participants were given oil/spread for *ad libitum* use in their cooking at home, which allowed them to share with their families, it was difficult to determine the exact amount they themselves actually consumed even though their average use was measured using a 3DDR. Second, for those who used the oil for frying, only some of the oil absorbed by the food was expected to be ingested. The plasma fatty acid analysis reflected this situation since it showed a moderate 2 % increase with a wide range (−3.84 % to 7.32 %) in plasma oleic acid levels after 3 months in the olive oil group compared to the control group.

Nevertheless, based on significant changes in plasma oleic acid as well as the information recorded by 3DDR, both groups (OO and CON) were compliant to the treatments. We are aware that measuring blood levels of phenolic compounds present in olive oil could have enhanced compliance validation and data interpretation. However, in the current study, the participants used the oils at home at different times, thus a pre-determined time between olive oil consumption and blood collection could not be tightly controlled. Given that these phenolic compounds are metabolized over time after absorption, it would be unreliable to use random blood sample to assess phenolic compounds following olive oil consumption [66].

In conclusion, our findings suggest that substituting EVOO for oils used in a typical American diet may have both cardio-metabolic and immunologic health benefits as indicated by reduced systolic blood pressure, a strong trend toward increased plasma HDL-C, and a moderate enhancing effect on T cell-mediated function. However, our results do not support an anti-inflammatory effect of EVOO in obese older adults. Given the increased risk of high blood pressure and a decline in T cell-mediated function in older adults, particularly those who are overweight and obese, our results suggest that overweight and obese older adults might benefit from substituting the oils used in their typical American diet with extra virgin olive oil.

## Abbreviations

CON: Control
OO: Olive oil
EVOO: Extra virgin olive oil
Med-diet: Mediterranean diet
CVD: Cardiovascular disease
HNRCA: Human Nutrition Research Center on Aging
MRU: Metabolic Research Unit
BMI: Body mass index
NHANES: National Health and Nutrition Examination Survey
DTH: Delayed-type hypersensitivity
CBC: Complete blood count
3DDR: 3-day dietary record
LDL-C: Low density lipoprotein cholesterol
HDL-C: High density lipoprotein cholesterol
GC: Gas chromatography
ConA: Concanavalin A
IL-2: Interleukin-2
IL-6: Interleukin-6
IFN-γ: Interferon-gamma
TNF-α: Tumor necrosis factor-alpha
PGE_2_: Prostaglandin E_2_
LPS: Lipopolysaccharide
ELISA: Enzyme-linked immunosorbent assay
EIA: Enzyme immune assay
MUFA: Monounsaturated fatty acids
PUFA: Polyunsaturated fatty acids.
